# The impact of physical fitness and body mass index in children on the development of acute mountain sickness: A prospective observational study

**DOI:** 10.1186/s12887-015-0373-0

**Published:** 2015-05-08

**Authors:** Shih-Hao Wu, Yin-Chou Lin, Yi-Ming Weng, Yu-Hui Chiu, Wen-Cheng Li, Shih-Hao Wang, Chang-Wei Chan, Te-Fa Chiu, Kuo-Feng Huang, Chung-Hsien Chen

**Affiliations:** Department of Emergency Medicine, Chang Gung Memorial Hospital at Linkou, Taoyuan, Taiwan; Chang Gung University School of Medicine, Taoyuan, Taiwan; Department of Physical and Rehabilitation Medicine, Chang Gung Memorial Hospital at Taoyuan, Taoyuan, Taiwan; Department of Emergency Medicine, Mackay Memorial Hospital, Taipei, Taiwan; Institute of Environmental and Occupational Health Science, National Yang-Ming University, Taipei, Taiwan; Department of Occupation Medicine, Chang-Gung Memorial Hospital at Keelung, Keelung, Taiwan; Department of Occupation Medicine, Chang-Gung Memorial Hospital at Linkou, Taoyuan, Taiwan; Department of Physical Medicine and Rehabilitation, Chang Gung Memorial Hospital at Chiayi, Chiayi, Taiwan; Department of Emergency Medicine, National Yang-Ming University Hospital, Yilan, Taiwan; Altitude Research Center, Department of Emergency Medicine, School of Medicine, University of Colorado Anschutz Medical Campus, Aurora, Colorado USA; Taiwan Wilderness Medical Association, Taipei, Taiwan; Department of Physical Education, National Taitung University, Taitung, Taiwan; Department of Emergency Medicine, Taiwan Adventist Hospital, Taipei, Taiwan; Department of Family Medicine, Chang Gung Memorial Hospital at Linkou, Taoyuan, Taiwan

**Keywords:** Physical Fitness, 3-minute step test, Body Mass Index, Acute Mountain Sickness, Children

## Abstract

**Background:**

Acute mountain sickness (AMS) is commonly found among people traveling above 2500 m. We investigated whether the occurrence of AMS is related to differences in individual physical fitness and BMI in subjects 11–13 years of age.

**Methods:**

This study was conducted at Xue Mountain, Taiwan (elevation of 3886 m) between June 13, 2011 and June 17, 2011. Subjects were asked to ascend from Taipei City (25 m) to the summit (3886 m) over 3 days and 2 nights. Gender, age, weight, height, and fitness index (determined using a 3-minute step test) were recorded at sea level before ascent. The Lake Louise AMS score was used to record symptoms and diagnose AMS.

**Results:**

A total of 179 subjects (mean age: 11.8 years; 102 males, 77 females) were included in the analysis. A total of 44.7% of subjects were diagnosed with AMS. Male gender (*p* = 0.004) and elevated body mass index (BMI) (*p* < 0.001) were each associated with the development of AMS. However the physical fitness index was comparable in subjects with and without AMS (67.8 ± 10.1 vs. 68.0 ± 9.3, *p =* 0.9).

**Conclusions:**

This study shows that both BMI and male gender were associated with the development of AMS in 11–13 year old children. Physical fitness was not associated with the occurrence of AMS.

**Electronic supplementary material:**

The online version of this article (doi:10.1186/s12887-015-0373-0) contains supplementary material, which is available to authorized users.

## Background

Acute mountain sickness (AMS) is a well-recognized phenomenon in people travelling to high altitude, and is characterized by symptoms of headache, nausea or vomiting, dyspnea, fatigue, poor appetite, dizziness, and difficulty sleeping [1]. AMS is especially prevalent at altitudes greater than 2500 m, [2] and its development in climbers is the reason for many mountain rescues [3-5].

An increasing number of children are traveling to high altitudes on school expeditions or school adventure holidays, and during family expeditions [2]. Because transportation to many high altitude expedition sites has become more accessible, people of all ages who might not otherwise have been exposed to high altitudes are now being exposed to high altitude conditions.

Few studies have been published that have examined the incidence of AMS in children and adolescents, although the incidence of AMS in adults traveling to high altitudes is well documented. In adults, the incidence of AMS has been reported to range from 25% (at 2975 m) up to 75% (at 5896 m) [2,6]. AMS has been recorded in both adults and children, but the relative incidence in children and adolescents compared to adults remains unclear, and it has been suggested that the incidence and severity of AMS may be inversely related to climbers’ age [7,8] Hackett et al. found younger trekkers were more susceptible to AMS [9], and Honigman et al. reported an incidence of 45% in subjects aged between 16 and 19 years (compared with an overall average of 25%) [10].

Many factors may contribute to the development of AMS, including age, gender, obesity, ascent rate, sleeping altitude, history of AMS, previous exposure to high altitude, individual susceptibility, and a history of cardiopulmonary disease [3,10-14]. A previous report in adults has suggested that physical fitness provides protection from AMS [15]. However, another study reported that poor physical fitness does not worsen AMS at simulated high altitudes [16].

The physiological response to high altitude in children has been previously examined, [2,17-21]; but the relationship between physical fitness and AMS in children or adolescents has not been elucidated. The current study was designed to investigate the relationship between AMS and physical fitness and BMI in children from Taiwan.

## Methods

### Participants

A total of 179 subjects (age range: 11–13 years) participated in this prospective analysis (102 males and 77 females). None of the subjects had a history of exposure to high altitude (>2500 m above sea level) in the previous 6 months. All subjects were healthy, lived at altitudes below 500 m, and were asked not to take any medication for high-altitude illness before climbing and exposure to high altitude. None of the study participants reported prior AMS. All subjects had participated for 9 months in training regimens including swimming for 20 minutes once a week, jogging for 15 minutes once a week, and undergoing a stepping exercise for 20 minutes once a week in their physical education class at school. All written informed consent was obtained from the participants and their parents.

### Study design

All subjects attended the same elementary school and were divided according to their previously determined class selection (3 classes). One month prior to high altitude exposure, each subject provided a complete medical history (including gender, age, AMS history, body weight, height, body mass index [BMI], and fitness index [using the 3-min step test]) [22]. Each subject was also given a complete physical examination. A physician performed the physical exam and trained coaches administered the 3-minute step test.

On June 13, 2011 (Day 1), the subjects ascended by bus from Taipei City (elevation 25 m) to the trailhead at Xue Mountain (2150 m) in approximately 6 h. Next, subjects were asked to walk from the trailhead to Qika Hut (elevation 2480 m) in approximately 2 hours. Once at Qika hut (Night 1), trained physicians performed a physical exam, as well as an AMS evaluation. On Day 2, subjects ascended to Sanliujiu Hut (3154 m) in approximately 6 hours. Once again, trained physicians performed a physical exam, as well as AMS scoring (Night 2). On Day 3, subjects ascended to the summit of Xue Mountain (3886 m) in approximately 5 h, followed by descent to Sanliujian Hut for a physical exam and an AMS evaluation (sleep status on night 2 was used in the scoring of the Day 3 evaluation). Subjects then continued their descent to the trailhead and were transported home. Each group departed 1 day apart from the previous group due to the limited maximal capacity at the Qika and Sanliujiu huts. In total, the study began on 13 June 2011 and was completed on 17 June 2011. All protocols and procedures were approved by Ethics Committee of the Chang Gung Memorial Hospital, and informed consent was obtained from all participants and their parents.

### Evaluation of AMS

The Lake Louise AMS questionnaire was used to record symptoms including headache, gastrointestinal symptoms (poor appetite, nausea, or vomiting), fatigue and/or weakness, dizziness or lightheadedness, and difficulty sleeping [23]. Briefly, all symptoms were scored on a scale from 0 to 3 indicative of no, mild, moderate, or severe symptoms, respectively. A diagnosis of AMS was made if headache was present and was accompanied by at least one other symptom for a total AMS score ≥ 3. Only those subjects met the criteria for AMS were administered treatments. Treatments included acetaminophen for pain associated with headache, primperan for vomiting, acetazolamine plus descent to low altitude for severe discomfort, breathlessness, loss of balance, and pulmonary/cerebral oedema, and a delayed or stopped ascent for discomfort with no pulmonary/cerebral oedema. Questions from the Lake Louise AMS questionnaire were asked by the physicians, not self-reported by the subjects. The reason that self-reporting was not used was because the authors, who are physicians who have worked with the school for several years, have found that children in the age group used in the study usually cannot complete the questionnaires seriously and give wrong answers just for fun. Therefore the authors used an adult authority combined with the physician’s clinical observations to complete the questionnaire.

### Three-minute step test

Maximal oxygen consumption (VO2max) attained during a graded exercise test to voluntary exhaustion is considered the standard in evaluating cardiorespiratory fitness, or the overall capacity of the cardiovascular and respiratory systems to perform prolonged strenuous exercise [24]. However, for the purposes of the current study design, factors including accessibility, convenience, and subject limitations, caused us to use the 3-minute step test instead of VO2max to evaluate physical fitness. Another reason to use the 3-minute step test was that in Taiwan the official fitness standard of the Ministry of Education is based on data using the 3-minute step test, and its use would allow for future comparisons within Taiwan.

In this fitness test, stepping is performed to raise subjects heart rate (HR), and data are recorded using a three-in-one 3-min step tester (ACC-750B, Accuratus International Health Company).The height of the step was 35 cm and a metronome was used for timing at a rate of 96 times/min. The details of this test are given in the website (http://www.accuratus.com.tw/upload/userfiles/140783380259414731.pdf). The rate of HR recovery following the stepping exercise is used as an indicator of cardiopulmonary fitness; the faster the HR returns to the resting HR, the better the cardiopulmonary fitness of the subject. Upon test completion, the subject immediately sat down, and HR was obtained using the sum of heart beats measured between 1 and 1.5 min (HR1), 2 and 2.5 min (HR2), and 3 and 3.5 min (HR3) after the end of the exercise.

### Fitness index

The fitness score (fitness index) was calculated as follows: [exercise time × 100]/[(HR1 + HR2 + HR3) × 2], where exercise time is the duration of the test. Interpretation of the degree of fitness was based on data from surveys of the Ministry of Education, Taiwan (http://www.accuratus.com.tw/upload/userfiles/140783380259414731.pdf and http://www.sces.hcc.edu.tw/ezfiles/15/1015/attach/30/pta_31555_3370885_18855.doc and http://homepage.vghtpe.gov.tw/~nutr/forum/forum02/bmi2.htm). The test was stopped immediately if the participant experienced exhaustion, lost balance, missed rhythm for three consecutive steps, or experienced any discomfort at any stage of the test. If a participant could not complete the 3-minute step test, the time of termination of the test was recorded and used for analysis. The exercise time was multiplied by 100 to produce an easily divisible number. The step tester was operated by coaches certified by the Aerobics and Fitness Association of America. The test was performed before lunch in the same classroom, at low altitude (88 m), and with air conditioning to maintain a temperature of approximately 23°C (73.4°F), approximately 1 month before exposure to high altitude. The medical examination was to have been performed 1 week before exposure in our original protocol, but the high altitude exposure activity was delayed for 3 weeks due to a typhoon. However subjects did not change their life style or have high altitude exposure during this delay.

### Statistical analysis

Comparisons between children with and without AMS were made using independent t-tests, Chi-square tests, and Fisher’s exact test. Subjects were divided into subgroups by fitness index and BMI, with the chi-square test used for comparisons. Multivariate logistic regression was used to estimate odds ratios (OR) and 95% confidence intervals (95%CI) for the effects of BMI, physical fitness, age, and gender on the likelihood of experiencing AMS. Differences were considered significant with a two-sided p-value < 0.05. All statistical analyses were performed using SAS 9.2 statistics software (SAS Inc., Cary, NC, USA). Continuous variables (age, body weight, body height, BMI, physical fitness index) were presented as mean and standard deviation (mean ± SD) and categorical variables were as n (%).

## Results

### Subject characteristics

**Figure 1:**
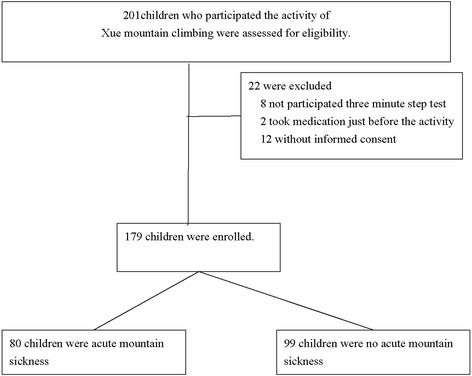
Study Design.

Figure 1 shows the selection process. Of the 201 children initially enrolled in the study, 179 (102 males, 77 females) were eligible for inclusion in the analysis. Baseline patient characteristics are shown in Table 1. More than half (97, 54.2%) of the subjects had excellent fitness, 38 (21.2%) had good fitness, 32 (17.9%) had fair fitness, and 12 (6.7%) had below-average fitness. No subject had poor fitness. There were no significant differences between the AMS and non-AMS groups in mean physical fitness index, age, or body height. The percentage of males, and the mean body weight, BMI, and BMI z-scores were all significantly higher in the AMS than in the non-AMS group. The percentage of females was lower in the AMS than in the non-AMS group.

**Table 1:** **Baseline characteristics between AMS and non-AMS groups**

**Patient characteristics**	**Total (N = 179)**	**AMS group (n = 80)**	**Non-AMS group (n = 99)**	***p value***
Gender				0.004*
Male, n (%)	102 (57%)	55 (68.8%)	47 (47.5%)	
Female, n (%)	77 (43%)	25 (31.2%)	52 (52.5%)	0.300
Mean age, years	11.8 ± 0.4	11.8 ± 0.5	11.8 ± 0.4	
Mean body weight, kg	45.0 ± 10.4	48 ± 11.8	42.6 ± 8.4	<0.001*
Mean body height, cm	152.0 ± 7.2	152.6 ± 7.8	151.5 ± 6.6	0.311
Mean body mass index^a^, kg/m2	19.4 ± 3.7	20.5 ± 4.2	18.4 ± 2.8	<0.001*
Mean BMI z-score^b^	2.8 ± 0.3	2.8 ± 0.2	2.7 ± 0.3	0.002*
Physical fitness index	67.9 ± 9.7	67.8 ± 10.1	68.0 ± 9.3	0.900

* Significantly different between AMS and non-AMS groups, respectively.

AMS, acute mountain sickness; BMI, body mass index.

^a^BMI = weight in kilograms divided by the square of the height in meters.

^b^BMI z-score was calculated as outlined by Center for Disease Control (CDC) guidelines (http://www.cdc.gov/growthcharts/percentile_data_files.htm).

Continuous variable was presented as mean ± SD, and compared the differences between with and without AMS using independent t-test.

Categorical variable was presented as count and percentage, and compared the differences between with and without AMS using Chi-square test.

Fourteen of the children included in the analysis did not complete the ascent of Xue mountain, due to several reasons, including a self-reported feeling of being uncomfortable, AMS, and higher AMS scores but not AMS itself. Eleven of the 14 were diagnosed with AMS.

### Incidence of AMS

The incidence of AMS was 6.1% on Night 1, 31.5% on 2, and 27% after summit on Day 3. Those with AMS were significantly more likely to be male (*p <* 0.004) and to have a high BMI (*p <* 0.001).

### Physical fitness and acute mountain sickness

**Figure 2:**
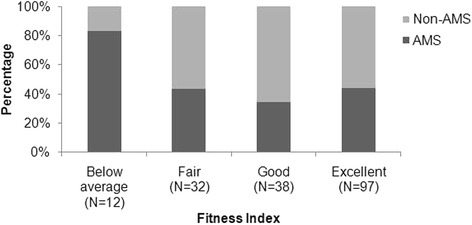
Incidence of AMS by subject fitness level. Derived by chi-square test for the comparison between AMS or no AMS and fitness level, *p*-value = 0.03.AMS, acute mountain sickness.

The standard used for assigning physical fitness levels is described in Additional file 1 Table 1. The mean physical fitness index of the AMS and non-AMS groups was similar (Table 1), and the mean physical fitness of those with AMS who did not complete the study was similar to that of AMS subjects who did complete the study (data not shown). Although the mean level of physical fitness was similar in both groups, more than 80% of the subjects in the lowest fitness category had AMS (Figure 2).

The incidence of AMS was significantly different among subjects across fitness index levels (*p =* 0.03), and compared with the non-AMS group, the incidence of AMS was lower in subjects with fair (43.8% vs. 56.3%, *p =* 0.48), good (34.2% vs. 65.8%, *p =* 0.052), and excellent (44.3% vs.55.7%, *p =* 0.264) fitness; and higher in those with below average fitness (83.3% vs. 16.7%, *p =* 0.021).

### Body mass index and acute mountain sickness

**Figure 3:**
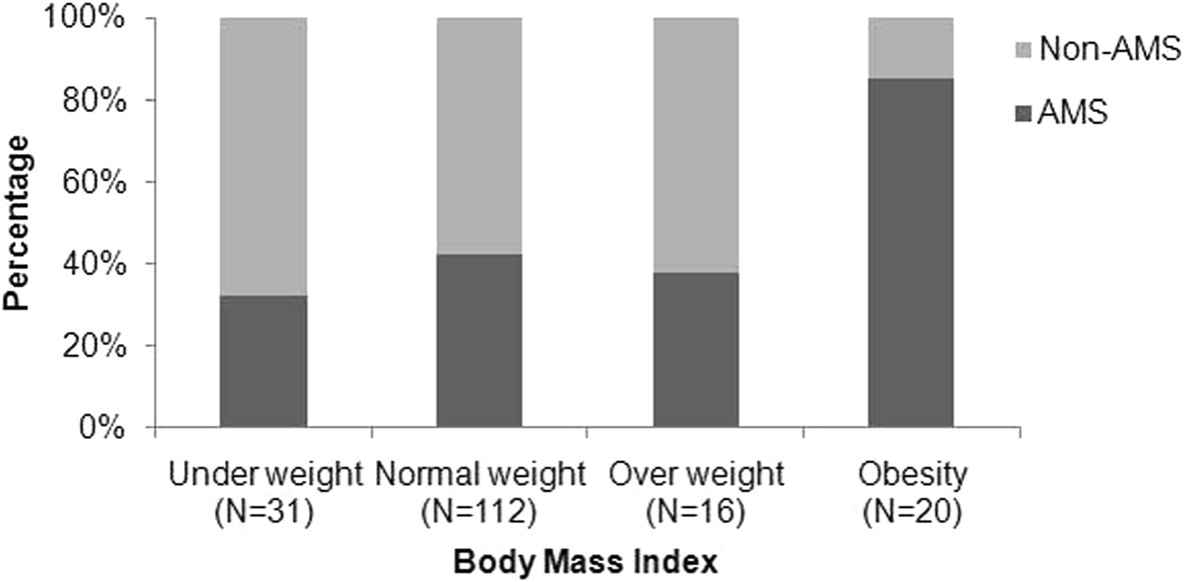
Incidence of AMS by subject BMI. Derived by chi-square test for the comparison between AMS or no AMS and BMI, *p*-value = 0.001.AMS, acute mountain sickness.

The standards for BMI are described in Additional file 1 Table 2. The incidence of AMS was significantly different across the range of BMI levels (*p* = 0.001; Figure 3) and was particularly high (85%) in the obese. The incidence of AMS compared to no AMS was lower in the 31 underweight (32.3% vs. 67.7%, *p =* 0.048), 112 normal weight (42.0% vs. 58.0%, *p =* 0.089), and 16 overweight (37.5% vs. 62.5%, *p =* 0.317) subjects. In the 20 obese subjects, the incidence of AMS was significantly higher than in those with no AMS (85% vs. 15%, *p =* 0.002).

**Table 2:** **Contributing factors to AMS (multivariable logistic regression)**

	**OR (95% CI)**	***p value***
**Model 1**		
Body mass index, kg/m2	1.18 (1.07 - 1.3)	0.001*
Age, years	0.73 (0.35 - 1.52)	0.403
Gender (Ref: Female)	2.37 (1.19 - 4.72)	0.014*
**Model 2**		
Body mass index z-score	6.68 (1.75 - 25.49)	0.006*
Age, years	0.68 (0.33 - 1.41)	0.298
Gender (Ref: Female)	2.53 (1.28 - 4.99)	0.008*
**Model 3**		
Physical fitness index	0.99 (0.96 - 1.02)	0.531
Age, years	0.71 (0.35 - 1.43)	0.335
Gender (Ref: Female)	2.78 (1.41 - 5.46)	0.003*

*Significantly different between AMS and non-AMS groups.

AMS, acute mountain sickness.

Multivariate logistic regression was used to estimate odds ratios (OR) and 95% confidence intervals (95%CI) for the effects of BMI, physical fitness, age, and gender on the likelihood of experiencing AMS.

### Body mass index and Physical fitness

As showed in Table 3, there were no significant correlation between BMI and physical fitness level in children with AMS (*p =* 0.0502).

**Table 3:** **The correlation of physical fitness level and BMI in AMS group**

	**Physical fitness level**				
	**Below average**	**Fair**	**Good**	**Excellent**	**p-value**
BMI					0.0502
Under weight	0 (0%)	2 (14.3%)	3 (23.1%)	5 (11.6%)	
Normal weight	4 (40%)	6 (42.9%)	9 (69.2%)	28 (65.1%)	
Over weight	0 (0%)	2 (14.3%)	1 (7.7%)	3 (7.0%)	
Obesity	6 (60%)	4 (28.6%)	0 (0%)	7 (16.3%)	

BMI, body mass index; AMS, acute mountain sickness.

Fisher’s exact test was performed to compare the correlation between BMI and physical fitness level.

### Multivariate logistic regression

Three multivariate logistic regression models were performed to determine the impact of specific factors on the risk of AMS (Table 2). In model 1, BMI, gender, and age were analyzed. The odds of AMS increased with increased BMI (OR = 1.18, *p* = 0.001). After adjusting for BMI and age the odds of AMS increased in males compared with females (OR = 2.37, *p* = 0.014). No significant difference was found between groups in terms of age (*p* > 0.05).

In model 2, BMI z-score, age, and gender were analyzed. The odds of AMS increased with increased BMI z-score (OR = 6.68, *p* = 0.006). After adjusting for BMI z-score and age, the odds of AMS increased in males compared with females (OR = 2.53, *p* = 0.008). No significant difference was found between groups in terms of age (*p* > 0.05).

In model 3, physical fitness index, age, and gender were analyzed. No significant differences were found between groups in terms of individual physical fitness index and age (*p* > 0.05). The odds of AMS were increased in males compared with females (OR = 2.78, *p* = 0.003).

## Discussion

The relationship between AMS, physical fitness, and obesity in children has not previously been studied. In the current study, male sex and BMI, but not physical fitness, were significantly and independently associated with AMS likelihood in children. However, although physical fitness had no significant association with risk of AMS the small group of children in the lowest physical fitness category had a higher percentage with AMS than was seen in groups with better fitness.

In our study, the physical fitness level did not affect the OR for AMS. Studies in adults of the relationship between physical fitness and AMS have given varying results. Good physical condition was reported to decrease the likelihood of AMS during an ascent from 1920 to 2957 m in the Rocky Mountains. [10] The development of AMS at 3000 m was reported to be unrelated to the level of habitual physical activity at sea level [25]. No correlation between physical fitness (measured by VO2max) and AMS was seen in trekkers with frequent exposure to high altitude who ascended to 4500 m over a 7 day period [15]. Physical fitness (measured by maximum oxygen consumption) was unrelated to development of AMS in a study of mountaineers who ascended to 4559 m [26]. It was also reported that elevated VO2max was associated with development of AMS at altitudes of 2400 to 5300 m studies in Alaska, Tibet, and Nepal [18]. However, there was a large overlap between AMS and non-AMS groups in the latter report.

These results from adult studies suggest a possible, although not probable, weak correlation between good physical condition and decreased likelihood of AMS. Our results in children do not suggest such a correlation, although the small number of children in the lowest physical fitness class did have an increased incidence of AMS. In interpreting these results, one must consider two things: (1) that many of the symptoms used to diagnose AMS (headache, nausea, poor appetite, dizziness, difficulty sleeping) are central nervous system symptoms, and have little relationship to cardiovascular fitness, and (2) that fatigue, the symptom that is related to both AMS and cardiovascular fitness, might have been pronounced in the children in the lowest fitness group, and its severity might have influenced the results. However, we have no sea level results for exercise of a similar magnitude and duration in poorly fit children, so we cannot answer this question.

In addition to gender, a high BMI was an independent risk factor for AMS. It needs to be noted that it was BMI itself, not any association of BMI with fitness, that increased the risk of AMS in this study. The OR for risk of AMS increased from 1.18 to 6.68 when the BMI-z score was used instead of BMI. The z score substitutes the standard deviation level of the individual BMI score for the actual BMI score, and thus increased the contribution of the group in our study with very high obesity and therefore high z-scores. This obese group, as can be seen in Figure 3, had a particularly high incidence of AMS.

The current finding that AMS is more common in boys than girls was somewhat surprising. In a small group of adolescents ascending to over 5500 m, Imray and colleagues found AMS to be more common in girls than boys [27]. In a slightly older group (mean age 15.9 years) Dallimore and Rowbotham also found that girls had a statistically higher incidence of AMS than boys [28]. Bloch and colleagues found no differences between the sexes, nor did Pradhan in 36 children ascending to 4380 m [17,21]. The difference between sexes in our study was not due to a difference in BMI, for no significant difference in BMI between boys and girls was observed. The association between gender and AMS development requires further investigation.

Young age may be associated with the development of AMS [20]. Wang et al. [4] reported age as a risk factor for the development of AMS, with a reported AMS prevalence of 36% in trekkers at Jade Mountain, Taiwan. We currently report an AMS incidence of approximately 45% and provide further evidence supporting a higher risk for AMS in children. However, lower AMS incidence rates have also been reported. Bloch and colleagues reported an AMS incidence of 37.5% in children ascending rapidly to 3450 m [17]. And Rehaj, in his study, reported AMS to be much more prevalent in adults (66%) than in children (22%), [29].

### Study limitations

Our study has several limitations. First, the study population was not wholly representative of the age-matched general population in Taiwan since study participants underwent fitness training prior to exposure to altitude, thus improving their overall fitness level. And we were unable, because of the study design, to choose 2 groups of children with clearly different levels of physical activity and compare the 2 groups for presence of AMS. Second, the sample size for subjects with below-average fitness was relatively small. Third, participants were permitted to stop the 3-minute step test upon onset of lost balance, missed steps, or discomfort; therefore, it is possible these results could have been impacted by low motivation or poor balance (although all students seemed enthusiastic and energetic during all fitness tests, and all participants for analysis completed the tests). Fourth, development of symptoms indicative of AMS may have been in part due to factors other than altitude exposure such as travel anxiety and lack of sleep [30]. Fifth, treatments were administered to several AMS subjects for symptom relief (acetaminophen, metoclopramide, or acetazolamide); if symptoms persisted, descent was suggested. Therefore, it is not possible to assess the worst AMS score among our study subjects, nor could further analysis of AMS severity be performed. Sixth, the physical status may change during the 3 weeks delay of high altitude exposure. Because subjects did not change their life style and did not have high altitude exposure during the delay, therefore the changes might be minimal. Finally, this study was performed in Taiwan with all Taiwanese subjects; therefore, interpretation of these results may not be transferrable to other populations.

## Conclusions

Consistent with previous data we currently report no relationship between fitness index and AMS. However, there was a higher incidence of AMS in subjects in the lowest fitness category, a majority of whom were males. Lastly, high BMI and male gender were positively associated with the development of AMS.

## Additional file

Additional file 1:
**Physical fitness standards of 11-13 year-old children.**

## Abbreviations

AMS: Acute mountain sickness
BMI: Body mass index
