# Supplementary material for: The impact of physical fitness and body mass index in children on the development of acute mountain sickness: A prospective observational study
Source: BMC Pediatr. 2015 May 8;15:55. doi: 10.1186/s12887-015-0373-0 (PMC4493965; doi:10.1186/s12887-015-0373-0)
Supplement: Additional file 1: — Physical fitness standards of 11-13 year-old children. [file 12887_2015_373_MOESM1_ESM.doc]

Supplemental Table 1. Physical fitness standards of 11-13 year-old children

| For male children | | | | | |
| --- | --- | --- | --- | --- | --- |
| Age | Poor | Below average | Fair | Good | Excellent |
| 11 | ~52.4 | 52.6-56.9 | 57.0-61.5 | 61.6-66.4 | 66.5~ |
| 12 | ~51.1 | 51.2-58.0 | 58.1-62.7 | 62.8-70.5 | 70.6~ |
| 13 | ~54.2 | 54.3-59.0 | 59.1-64.1 | 64.2-69.7 | 69.8~ |
| For female children | | | | | |
| Age | Poor | Below average | Fair | Good | Excellent |
| 11 | ~51.3 | 51.4-54.3 | 54.4-58.3 | 58.4-64.8 | 64.9~ |
| 12 | ~48.7 | 48.8-53.2 | 53.3-56.0 | 56.1-61.5 | 61.6~ |
| 13 | ~48.4 | 48.5-53.2 | 53.3-57.6 | 57.7-64.5 | 64.6~ |

Supplemental Table 2. BMI standards of 11-13 year-old children

| For male children | | | | |
| --- | --- | --- | --- | --- |
| Age | Under weight | Normal weight | Over weight | Obesity |
| 11 | ≤15.8 | 15.8-21.0 | 21.0-23.5 | ≥23.5 |
| 12 | ≤16.4 | 16.4-21.5 | 21.5-24.2 | ≥24.2 |
| 13 | ≤17.0 | 17.0-22.2 | 22.2-24.8 | ≥24.8 |
| For female children | | | | |
| Age | Under weight | Normal weight | Over weight | Obesity |
| 11 | ≤15.8 | 15.8-20.9 | 20.9-23.1 | ≥23.1 |
| 12 | ≤16.4 | 16.4-21.6 | 21.6-23.9 | ≥23.9 |
| 13 | ≤17.0 | 17.0-22.2 | 22.2-24.6 | ≥24.6 |
